# Soluble E-Cadherin as a New Prognostic Biomarker Able to Predict Survival in Newly Diagnosed Diffuse Large B-Cell Lymphoma

**DOI:** 10.3390/biom16050637

**Published:** 2026-04-24

**Authors:** Paola Carolina Rabesquini Marcelino da Silva, Igor Cerejo Tavares da Silva de Almeida, Joaquim Gasparini dos Santos, Leonardo Jun Otuyama, Cadiele Oliana Reichert, Hebert Fabrício Culler, Nélio Cézar de Aquino, Lilian de Souza França, Sheila Aparecida Coelho da Siqueira, Renata de Oliveira Costa, Vanderson Rocha, Sérgio Paulo Bydlowski, Débora Levy, Juliana Pereira, Luís Alberto de Pádua Covas Lage

**Affiliations:** 1 Laboratory of Medical Investigation in Pathogenesis and Directed Therapy in Immuno-Onco-Hematology (LIM-31), Faculty of Medicine, University of Sao Paulo, Sao Paulo 05430-000, Brazil; paola.marcelino@usp.br (P.C.R.M.d.S.); i.cerejo@hc.fm.usp.br (I.C.T.d.S.d.A.); cadiele.reichert@hc.fm.usp.br (C.O.R.); hebert.culler@hc.fm.usp.br (H.F.C.); nelio.aquino@alumini.usp.br (N.C.d.A.); lilian.sfranca@hc.fm.usp.br (L.d.S.F.); vanderson.rocha@hc.fm.usp.br (V.R.); juliana.pereira@hc.fm.usp.br (J.P.); 2 Department of Hematology, Hemotherapy & Cell Therapy, Faculty of Medicine, University of Sao Paulo, Sao Paulo 05430-000, Brazil; joaquim.gasparini@hc.f.usp.br (J.G.d.S.); leonardo.otuyama@hc.fm.usp.br (L.J.O.); spbydlow@usp.br (S.P.B.); 3 Department of Pathology, Faculty of Medicine, University of Sao Paulo, Sao Paulo 05430-000, Brazil; sheila.siqueira@hc.fm.usp.br; 4 Department of Hematology, Faculdade de Ciências Médicas de Santos, Fundação Lusíada, Santos 11050-071, Brazil; renatadeoliveiracosta@uol.com.br; 5 Department of Hematology & Oncology, Hospital Alemão Oswaldo Cruz, Sao Paulo 01323-020, Brazil; 6 Pró-Sangue Foundation, Blood Bank of Sao Paulo, Sao Paulo 05430-000, Brazil; 7 Department of Hematology, Churchill Hospital, Oxford University, Oxford OX12JD, UK; 8 Laboratory of Medical Investigation in Immunology and Histocompatibility (LIM-19), Faculty of Medicine, University of Sao Paulo, Sao Paulo 05430-000, Brazil; d.levy@hc.fm.usp.br

**Keywords:** diffuse large B-cell lymphoma, cell adhesion molecules, sICAM-1, sVCAM-1, sP-selectin, sE-cadherin, prognosis, cancer biomarkers

## Abstract

**Background:** Diffuse large B-cell lymphoma (DLBCL) is the most common subtype of non-Hodgkin lymphoma, constituting an important public health problem. Although curable, it presents a widely variable prognosis. The main tool used for prognostic stratification in DLBCL is the International Prognostic Index (IPI), which does not consider crucial biological variables for understanding its prognostic heterogeneity. Cell adhesion molecules (CAMs) play a central role in cancer biology and can be evaluated in affected tissues or in plasma, in soluble forms (sCAMs). CAMs promote proliferation, survival, and dissemination of malignant cells. Although extensively studied in solid tumors, their role remains unclear in hematological malignancies, particularly in DLBCL. **Methods:** This is a prospective and longitudinal study involving 87 newly diagnosed DLBCL (ND-DLBCL) patients aiming to quantify plasma levels of sCAMs (sICAM-1, sVCAM-1, sP-selectin, and sE-cadherin) at diagnosis and assessing its potential prognostic impact, as well as establishing clinical-biological associations. **Results:** Plasma quantification of sICAM-1, sVCAM-1, and sP-selectin did not present prognostic impact in DLBCL. However, continuous increases in sE-cadherin levels, as well as sE-cadherin ≥ 126.55 ng/mL were associated with lower response rates to R-CHOP regimen, higher frequency of recurrence following first-line therapy, and shortened survival. Additionally, sE-cadherin concentration ≥ 126.55 ng/mL was an independent predictor related to decreased overall survival. **Conclusion:** sE-cadherin measured at diagnosis has emerged as a new prognostic biomarker able to predict response, relapse and survival in ND-DLBCL.

## 1. Introduction

Diffuse large B-cell lymphoma (DLBCL) is an aggressive malignant neoplasm characterized by proliferation of large, atypical, and pleomorphic B-cells that diffusely infiltrate the affected tissue, disrupting its usual architecture [1,2]. It constitutes the most common subtype of non-Hodgkin lymphoma (NHL), accounting for 30–40% of all cases [2]. NHLs are an important public health problem, corresponding to the ninth most common cancer in the US and Europe and responsible for being the fifth cause of death due to malignancies [3]. Global statistics show the occurrence of 545,000 new NHL cases per year, being responsible for 260,000 deaths [3,4].

The prognosis of DLBCL is highly heterogeneous and dependent on clinical-laboratory, pathological, phenotypic, imaging, and genetic-molecular characteristics [2,5,6]. Currently, the most widely used tool to stratify prognosis and predict survival in DLBCL is the International Prognostic Index (IPI). IPI is based on the analysis of five independent prognostic variables, including age ≥ 60 years, elevated serum lactate dehydrogenase levels, Ann Arbor clinical stage III or IV, involvement of more than one extranodal site, and performance status by ECOG ≥ 2. According to IPI, patients are stratified into four categories with 3-year overall survival (OS) probability ranging from 91% for low-risk cases to only 59% in high-risk strata [7]. However, IPI has some limitations and does not incorporate biological variables able to reflect the prognostic heterogeneity of DLBCL [8].

In this sense, it was demonstrated that concomitant expression of MYC and BCL-2 proteins assessed by immunohistochemistry (*double-expressor lymphomas*), rearrangements involving *c-MYC* and *BCL-2* and/or *BCL-6* genes detected by fluorescent in situ hybridization (*double/triple hit high-grade B-cell lymphomas*), activated B-cell (ABC) phenotype determined by gene expression profile, and molecular signatures characterized by recurrent mutations involving *MYD88*, *CD79b*, and *NOTCH1* genes are associated with decreased OS [2,9,10,11]. Currently and accordingly to the WHO-HAEM 5th classification, high-grade B-cell lymphomas (HGBCL) containing *c-MYC* and *BCL-2* and/or *BCL-6* gene rearrangements constitute a separate entity, distinct from DLBCL, NOS. These malignancies present more aggressive biological behavior, inferior clinical outcomes, poor response to R-CHOP regimen and need intensified therapeutic approaches, commonly based on Burkitt-like chemotherapy protocols [12]. The composition of tumor microenvironment also influences DLBCL prognosis. Different studies associate the presence of fibrosis, high blood vessel density, dense macrophage tissue infiltrate, M2 histiocytic polarization, and “*dark zone*”-type macrophage transcriptomic signatures with poor clinical outcomes [2,13,14].

However, DLBCL prognostic stratification based on such genetic-molecular parameters usually has limited applicability in clinical practice. These tests involve sophisticated and high-cost technologies, result in delays in data acquisition, are not available in many regions of the world, have low reproducibility, and their interpretation depends on highly qualified professionals. Therefore, identification of plasma prognostic biomarkers in DLBCL is necessary, preferably using low-cost, widely available, highly reproducible, and rapid execution methodologies, such as plasma quantification of proteins potentially associated with tumor biology by enzyme-linked immunosorbent assay (ELISA).

Cell adhesion molecules (CAMs) constitute a group of transmembrane proteins responsible for cell adhesion to other cells or to the extracellular matrix. CAMs play a central role in leukocyte adhesion and transmigration through vascular endothelium, promoting interaction in inflammatory or tumor sites. CAMs are categorized into five groups, including integrins, selectins, cadherins, the immunoglobulin superfamily (IgSF), and mucins [15,16]. Their expression can be investigated in affected tissues using immunohistochemistry, and can also be quantified in plasma, in soluble forms, using simple methodologies such as ELISA or Western blot.

Among the main CAMs, ICAM-1, VCAM-1, P-selectin, and E-cadherin stand out. ICAM-1 (CD54) is an intracellular adhesion molecule, part of IgSF, expressed by fibroblasts, keratinocytes, endothelial cells, and leukocytes. ICAM-1 regulates leukocyte trafficking and diapedesis, serves as a co-stimulatory receptor for T cell activation, and is the main ligand for β2 integrins. Vascular cell adhesion molecule (VCAM-1) or CD106 is also part of IgSF, being predominantly expressed by endothelial cells, but under inflammatory conditions, it can be expressed by other cells. VCAM-1 is an important mediator of leukocyte rolling, adhesion, and extravasation, binding to α4β1 integrins. P-selectin or CD62P belongs to the subfamily of ionized calcium-dependent lectins, being predominantly expressed by platelets and endothelial cells. P-selectin participates in the initial capture of leukocytes to endothelium during leukocyte migration. E-cadherin, on the other hand, is a protein that forms calcium-dependent homophilic cell junctions. The loss of its function is associated with epithelial–mesenchymal cell transition, favoring cell migration capacity, motility, and invasiveness [15,16].

CAMs play an important biological role in cancer, being associated with the pathophysiology of both, solid tumors and hematological malignancies [16]. In solid tumors, they act by promoting the trafficking of leukocytes through bloodstream to tumor sites and maintain cohesion of neoplastic cells during the tumor dissemination process. CAMs promote growth and survival of malignant cells (mucins), participate in tumor progression and dissemination favoring cell proliferation, survival, migration, and tissue invasion (integrins), and act in epithelial–mesenchymal transition (cadherins), favoring tumor metastasis [15,16].

Malignant cell proliferation mediated by CAMs occurs when integrins bind to the extracellular matrix and activate pro-proliferative pathways, such as MAP/ERK. E-cadherin loss of function, commonly found in tumors, leads to the release of β-catenin into the cytoplasm, which then migrates to the nucleus and activates proliferative genes. Resistance to apoptosis, promoting tumor cell survival, occurs through integrin-mediated adhesion, leading to the activation of the anti-apoptotic PI3K/Akt pathway. Moreover, the formation of tumor cell clusters mediated by ICAM-1 protects these cells against mechanical stress and favors their immune evasion. CAMs also play a complex role in metastasis formation, acting in the epithelial–mesenchymal transition (E-cadherin) and favoring tissue invasiveness; selectins assist tumor cells in adhering to and rolling along the walls of blood vessels; also, the expression of some CAMs by tumor cells can favor the mimicry of such cells with leukocytes, favoring the free circulation of malignant cells in the bloodstream and allowing them to evade the immune system [15,16].

The role of CAMs in hematological malignancies is less defined, particularly in DLBCL. Few studies point to the action of different integrins promoting migration of neoplastic cells from bone marrow to secondary lymphoid organs in chronic lymphocytic leukemia, as well as an association between expression of the LFA-1 integrin and more aggressive disease and greater therapeutic resistance in multiple myeloma [17,18,19].

Considering the important role of CAMs in cancer biology, the possibility of quantifying them in plasma using simple and low-cost methodologies, and the need to identify prognostic biomarkers in DLBCL to refine its prognostic stratification and promote therapeutic strategies adapted to biological risk, we proposed to evaluate the potential prognostic impact of CAMs in this neoplasm. Therefore, we conducted a study aiming to quantify plasma levels of sICAM-1, sVCAM-1, sP-selectin, and sE-cadherin in newly diagnosed DLBCL (ND-DLBCL) and to seek associations between plasma quantification of these biomolecules with clinical outcomes, response to first-line therapy based on R-CHOP, and clinical-phenotypic variables.

## 2. Methods

### 2.1. Study Design, Population and Ethical Issues

A prospective, longitudinal and single-center study was conducted at Instituto do Câncer do Estado de São Paulo (ICESP), Hospital das Clínicas, Faculty of Medicine, University of Sao Paulo (FMUSP), Brazil, involving 87 ND-DLBCL patients, included between April 2021 and January 2025. The study obtained ethical approval from the local Ethics Committee (number 71016423.5.0000.0068). All research subjects included in the study gave their consent to participate by signing an Informed Consent Form (ICF). After ethical approval, clinical-demographic, laboratory, pathological, imaging, and therapeutic baseline data were extracted from electronic medical records and recorded on a *Research Electronic Data Capture* (Redcap) platform, as well as the results of experimental tests. The identification of research subjects was decoded in order to maintain anonymity, and only researchers involved in the study had access to the database.

### 2.2. Eligibility Criteria

The study included ND-DLBCL patients according to the criteria proposed by the World Health Organization Classification of Lymphoid and Hematopoietic Tissue Neoplasms published in 2022 (WHO HAEM 5th version) [2]. The following clinic-pathological variants were considered eligible: diffuse large B-cell lymphoma, not otherwise specified (DLBCL, NOS); Epstein–Barr virus-associated DLBCL (EBV+ DLBCL, NOS); primary cutaneous DLBCL, leg-type; DLBCL rich in T-cells/histiocytes (DLBCL rich T/H); ALK-positive DLBCL; and HHV8-associated DLBCL, NOS. Patients with double/triple expressor DLBCL, NOS were also included. Inclusion criteria also considered age ≥ 18 years, ICF signature, as well as diagnosis, treatment and follow-up at the Non-Hodgkin Lymphoma Outpatient Clinic of ICESP/HC-FMUSP.

Patients diagnosed with high-grade B-cell lymphoma with *c-MYC*, *BCL-2* and/or *BCL-6* gene rearrangements (double/triple hit lymphomas), primary mediastinal B-cell lymphoma (PMBCL), high-grade B-cell lymphomas of immunoprivileged sites (primary central nervous system lymphoma and primary testicular lymphoma), and immunosuppression-related B-cell lymphoproliferative disorders, including AIDS-related lymphomas (ARL) and post-transplant lymphoproliferative disorders (PTLD), were excluded. Additionally, patients with pre-existing inherited or acquired immunodeficiencies, synchronous solid tumors, cases previously exposed to radiotherapy, or undergoing corticosteroid therapy, cytoreductive chemotherapy, or any therapeutic intervention aimed at controlling lymphoma applied within four weeks preceding the collection of biological samples were also excluded.

### 2.3. Histopathological Diagnosis

All patients had a biopsy-confirmed diagnosis of DLBCL and met the criteria proposed by the fifth version of World Health Organization Classification [2]. Two experts in Hematopathology confirmed the diagnosis after independent analysis. Histopathological diagnosis was centered on morphological analysis using Hematoxylin-Eosin (HE) staining and immunohistochemical study based on a panel containing the markers Ki-67 (Dako, Carpinteria, CA, USA, J55, 1/1600), CD20 (Dako, L26, 1/1000), CD3 (Dako, F7.2.38, 1/500), CD5 (Invitrogen, Waltham, MA, USA, 53-7.3, 1/200), BCL-2 (Sigma-Aldrich, Darmstadt, Germany, B3170, 1/500), MYC (Invitrogen, 9E11, 1/200), CD10 (Novocastra, Nussloch, Germany, S6C6, 1/200), BCL-6 (Abcam, Cambridge, UK, EPR11410-43, 1/500), MUM-1/IRF4 (Abcam, EPR5653, 1/500), and ISH for EBV (*ZytoFast Plus CISH© kit*, ZytoVision, Bremerhaven, Germany). The markers CD10, BCL-6, and MUM-1/IRF4 were used to determine DLBCL cell of origin and categorize into germinal center B-cell (GCB) or non-germinal center B-cell (non-GCB) phenotype according to the immunohistochemical algorithm proposed by Hans et cols. [20]. In situ hybridization with fluorescent probes for *c-MYC* (break-apart Sure FISH MYC BA P20, Agilent Technologies, Santa Clara, CA, USA), *BCL-2* (break-apart Sure FISH BCL2 BA P20, Agilent Technologies, CA, USA), and *BCL-6* (break-apart Sure FISH BCL6 BA G111422, Agilent Technologies, CA, USA) was performed in all cases to exclude double/triple hit high-grade B-cell lymphomas.

### 2.4. Staging, Therapy and Response Assessment

Following diagnostic confirmation, all patients underwent staging procedures based on blood count evaluation, biochemical tests, including uric acid levels, electrolytes, renal function, liver tests, and baseline lactate dehydrogenase levels (LDH). Viral serologies for HIV and hepatitis B and C, as well as myocardial function assessment by transthoracic echocardiography, were part of the initial evaluation. Clinical staging, according to Lugano criteria (2014), was determined by computed tomography scans of the neck, thorax, abdomen, and pelvis integrated with 18-fluorodeoxyglucose PET-scans (PET-CT) [21]. Endoscopic evaluation of the gastrointestinal tract, magnetic resonance imaging of the central nervous system (CNS), and cerebrospinal fluid analysis were performed in selected cases, frequently guided by clinical evidence of involvement of these extranodal sites. As all cases were staged with 18-FDG-PET-CT, bone marrow biopsy was omitted from the staging procedures.

Initial therapy was based on the use of R-CHOP immunochemotherapy (rituximab 375 mg/sqm IV on day 1, cyclophosphamide 750 mg/sqm IV on day 1, doxorubicin 50 mg/sqm IV on day 1, vincristine 1.4 mg/sqm [maximum 2.0 mg] IV on day 1, and prednisone 100 mg/day PO on days 1 to 5) for a minimum of four and a maximum of eight cycles administered at 21-day intervals. Patients with ECOG ≥ 3 or significant comorbidities received 50% of doxorubicin dose (25 mg/sqm) in the first cycle, with the possibility of dose adjustment to 100% in subsequent cycles depending on improvement in performance status. Patients aged 80 years or older received R-miniCHOP protocol (rituximab 375 mg/sqm IV on day 1, cyclophosphamide 400 mg/sqm IV on day 1, doxorubicin 25 mg/sqm IV on day 1, vincristine 1.0 mg fixed dose IV on day 1, and prednisone 40 mg/sqm PO on days 1 to 5) for a maximum of 6 cycles.

Involved field radiotherapy (IF-RT) with 30–36 Gy was employed in early-stage disease (I or II) treated with 4 cycles of immunochemotherapy, in early-stage disease with bulky ≥ 7 cm, and in advanced-stage disease (III or IV) containing bulky with localized residual 18-FDG uptake at the end of immunochemotherapy. Cases presenting involvement of paranasal sinuses, breast, testes or ovaries, kidneys, adrenal glands, paravertebral mass with foramina invasion, and those with involvement of two or more extranodal sites and IPI ≥ 3 received CNS chemoprophylaxis with at least 4 intrathecal injections of methotrexate 12 mg plus dexamethasone 2 mg and/or two to three cycles of high-dose methotrexate at the end of immunochemotherapy (3000 mg/sqm IV every 15–21 days).

Response assessment followed the Lugano criteria [21], based on clinical, laboratory, and imaging parameters, including PET-CT interim (after the fourth chemotherapy cycle) and at the end of treatment (after the sixth or eighth cycles). Once complete remission was achieved, patients were regularly evaluated with clinical and laboratory tests during the first five years of remission.

### 2.5. Soluble CAMs (sCAMs) Quantification by Elisa

#### 2.5.1. Sample Collection and Processing

After signing the ICF, 8 mL of peripheral blood samples were collected from each participant in two tubes with ethylenediaminetetraacetic acid (EDTA). Initially, automated blood counts were performed using a Sysmex XN-550 analyzer (Sysmex Corporation, Kobe, Japan). Subsequently, the samples were centrifuged to separate the plasma, which was aliquoted into five 1.5 mL microtubes and stored in a freezer at −80 °C.

#### 2.5.2. Sandwich Enzyme-Linked Immunosorbent Assays

After thawing, plasma samples from 87 ND-DLBCL patients were processed for quantification of sCAMs using a sandwich enzyme-linked immunosorbent assay (sandwich ELISA). Commercial kits used were BMS201 (Invitrogen, Carlsbad, CA, USA) for sICAM-1, BMS232 (Invitrogen, Carlsbad, CA, USA) for sVCAM-1, BMS219-4 (Invitrogen, Carlsbad, CA, USA) for sP-selectin, and E-EL-H0014 (Elabscience, Wuhan, China) for sE-cadherin. Quantification of each sCAM by ELISA followed the manufacturer’s recommendations. In summary, the reagents were initially diluted and reconstituted. Subsequently, a standard curve was constructed through serial dilutions of the standard solution. Then, samples from each patient, standard solution, blank control, and positive controls were added to 96-well microplates. After washing and dilution, the conjugate was added, and the plates were incubated at room temperature. After incubation, the microwells were emptied, and the respective substrates were added. Reaction lectures were taken on an automated Elx808 analyzer (Agilent Biotechnologies, Santa Clara, CA, USA) after addition of stopping solution, and absorbance was measured at a wavelength of 450 nm.

### 2.6. Statistical Analysis

For descriptive statistics, continuous variables were presented as measures of central tendency (mean and median), dispersion (IqR 25–75%), and position. Categorical variables were described in absolute values (*N*) and percentages (%), with respective ranges (min-max) and 95% confidence intervals (95% CI). Overall survival (OS) and event-free survival (EFS) were determined using the Kaplan–Meier method, with construction of the respective survival curves. OS was defined as the time interval between DLBCL diagnosis and death from any cause or date of the last follow-up. EFS was defined as the time interval between diagnosis and date of relapse, progression, or death from any cause.

Analysis to determine outcome predictors, including those related to OS and EFS, was performed using Cox’s univariate semiparametric method. Multivariate analysis using Cox’s multi-step regression model was conducted to identify independent prognostic variables. Variables with a *p*-value ≤ 0.10 identified in univariate analysis were included in the final model for multivariate analysis, restricted to a maximum of ten variables given sample limitation.

Determination of cut-offs (CO, M-value) for each sCAM able to discriminate clinical outcome (OS and EFS) was performed using the maxstat test. After determining a statistically significant M-value (*p* ≤ 0.05), survival curves were constructed using the Kaplan–Meier method. Association between the median quantification values of each sCAM and the variables response to upfront therapy, relapse/progression, and death was performed using Wilcoxon test with Bonferroni correction. Association between the CO of each sCAM, and clinical-phenotypic and laboratory characteristics was performed using Wilcoxon test and qui-square or Fisher’s exact test.

Results were presented as hazard ratio (HR), 95% confidence interval (95% CI), and *p*-value. A *p*-value ≤ 0.05 was considered statistically significant. All analyses were performed using *RSoftware 4.3.0*, *RStudio 2022.12.0*, and *GraphPad Prism 10.6.1*.

## 3. Results

### 3.1. Baseline Clinical-Demographic, Laboratory and Pathological Features

Eighty-seven patients with biopsy-proven diagnosis of DLBCL were included in the study. The median age at diagnosis was 63 years (IqR 25–75%: 51–69), and 53% (46/87) were female. Eighty-three patients (95.4%) were classified as DLBCL, NOS; 2 (2.4%) as DLBCL rich T/H; 1 (1.1%) as DLBCL leg-type; and 1 (1.1%) as EBV-associated DLBCL, NOS. Using the immunohistochemical algorithm proposed by Hans et cols. 55% (48/87) were categorized as GCB phenotype and 45% (39/87) as non-GCB phenotype. Thirty-one patients (36%) presented early-stage disease (Ann Arbor I or II), and 56/87 (64%) presented advanced-stage disease (III or IV). Extranodal involvement was observed in 83% (72/87) of cases, with 45% (39/87) presenting involvement of two or more extranodal sites. Bulky disease ≥ 7 cm occurred in 47% (41/87) of cases, B-symptoms in 54% (47/87), 19% (16/87) presented bone marrow infiltration, and 3.4% (3/87) presented secondary CNS infiltration. Additionally, 23% (20/87) had poor performance status, categorized as ECOG ≥ 2.

Regarding prognostic stratification, 26% (23/87) of cases were categorized as low risk by IPI, 25% (22/87) as low-intermediate, 24% (21/87) as high-intermediate, and 24% (21/87) as high risk. According to R-IPI, 7% (6/87) of cases were stratified as very good (zero factors), 45% (39/87) as good (1–2 factors), and 48% (42/87) as poor (3–5 risk factors). All baseline clinical-epidemiological and pathological characteristics of the cohort are displayed in Table 1.

Median baseline values of hemoglobin, WBC, neutrophils, lymphocytes, monocytes, and platelets were 12.2 g/dL (IqR 25–75%: 10.7–14.0), 7.35 × 10^9^/L (IqR 25–75%: 5.46–9.51), 4.71 × 10^9^/L (IqR 25–75%: 3.38–6.65), 1.64 × 10^9^/L (IqR 25–75%: 1.05–2.32), 0.57 × 10^9^/L (IqR 25–75%: 0.41–0.82), and 277 × 10^9^/L (IqR 25–75%: 206–307), respectively. Additionally, median values of neutrophil/lymphocyte ratio and lymphocyte/monocyte ratio were 2.70 (IqR 25–75%: 1.90–4.50) and 2.64 (IqR 25–75%: 1.75–4.42), respectively. Baseline laboratory features presented as categorical variables are summarized in Table 2.

Median baseline values for sCAMs were 609 ng/mL (IqR 25–75%: 441–828) for sICAM-1, 4216 ng/mL (IqR 25–75%: 2696–5524) for sVCAM-1, 106 ng/mL (IqR 25–75%: 80–132) for sP-selectin, and 110 ng/mL (IqR 25–75%: 58–160) for sE-cadherin.

### 3.2. Therapy Patterns, Response and Mortality Rates

Among 87 ND-DLBCL patients included in the study, 84 (96.6%) were effectively treated with immunochemotherapy, while three patients (3.4%) did not experience any antineoplastic therapy because they died prematurely, before completing staging procedures. Sixty-four cases (76%) received full-dose R-CHOP regimen, 10 patients (12%) were treated with dose-adjusted R-CHOP (50% of doxorubicin dose), 5 individuals (6%) received R-miniCHOP, 3 patients (3.5%) received R-CHOMP (R-CHOP plus methotrexate 1500 mg/sqm IV on D1) because they presented secondary CNS infiltration, and 2 cases (2.5%) received R-CHOEP (R-CHOP plus etoposide 100 mg/sqm IV on D1-D3). Additionally, IF-RT was employed in 24% (20/84) of DLBCL patients, 34% (29/84) experienced cytoreduction with CVP (cyclophosphamide 300 mg/sqm IV on D1, vincristine 1.0 mg/sqm [maximum 2.0 mg] IV on D1, and prednisone 40 mg/sqm PO D1 to D7) for one cycle prior to full-dose immunochemotherapy beginning, and 37% (31/84) were submitted to CNS prophylaxis based on methotrexate and dexamethasone intrathecal injections.

The overall response rate (ORR) was 84% (95% CI: 74–90%), with complete response (CR) observed in 80% of cases (95% CI: 70–87%). The rate of refractoriness to first-line therapy based on R-CHOP-like regimens was 13% (95% CI: 7–22%). Relapse/progression rate was 10% (95% CI: 5–20%). Early mortality, i.e., that was observed in the first 100 days after the beginning of therapy, was observed in 6% (95% CI: 3–15%), while overall mortality rate (OMR), verified throughout the entire follow-up time, was 25% (95% CI: 17–36%). Among 22 deaths observed, 45% (10/22) were attributed to disease progression, 41% (9/22) resulted from infectious complications, and 14% (3/22) had unknown cause, as they occurred outside the service of origin.

### 3.3. Clinical Outcomes

**Figure 1:**
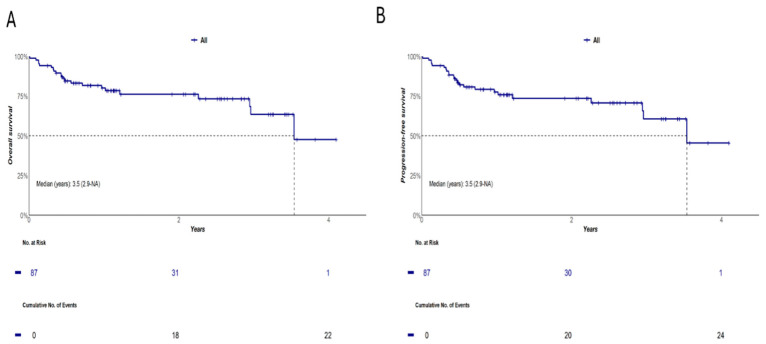
OS (**A**) and EFS (**B**) in 87 DLBCL patients included in the study.

With a median follow-up time of 1.09 years (95% CI: 0.79–1.21), both median OS and EFS were 3.5 years (95% CI: 2.9—not reached). Two-year and 4-year OS probabilities for 87 ND-DLBCL patients included in the study were 76% (95% CI: 67–87%) and 48% (95% CI: 26–88%), respectively. Similarly, 2-year and 4-year EFS probabilities were 74% (95% CI: 64–85%) and 45% (95% CI: 24–85%), respectively (Figure 1).

### 3.4. Prognostic Significance of sCAM Quantification in ND-DLBCL

Using maxstat test, discriminatory cut-off points for OS and EFS for sICAM-1, sVCAM-1, sP-selectin, and sE-cadherin were 942.65 ng/mL, 7001.35 ng/mL, 138.37 ng/mL, and 126.55 ng/mL, respectively. However, there was no statistical significance in determining M values for sICAM-1 (M = 2.540; *p* = 0.502), sVCAM-1 (M = 2.486; *p* = 0.603), and sP-selectin (M = 2.177; *p* = 1.0). Cut-off for sE-cadherin showed statistical significance for discriminating clinical outcomes using the maxstat test, with M = 3.383 and *p* = 0.034.

**Figure 2:**
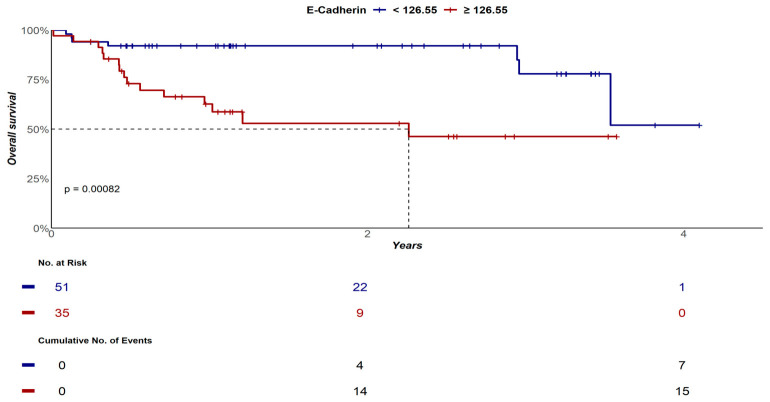
OS curves of DLBCL patients according to sE-cadherin quantification at diagnosis.

Based on this premise, the median OS for DLBCL patients containing pre-treatment plasma sE-cadherin levels < 126.55 ng/mL was not reached (95% CI: 3.5 years—not reached), while the median OS for individuals with sE-cadherin quantification ≥ 126.55 ng/mL was 2.3 years (95% CI: 0.9—not reached), *p* < 0.001. OS probabilities at 2 years and at 4 years for patients with sE-cadherin < 126.55 ng/mL were 92% (95% CI: 85–100%) and 52% (95% CI: 23–100%), respectively; while OS probabilities at 2 years and at 4 years for patients with sE-cadherin ≥ 126.55 ng/mL were 53% (95% CI: 37–76%) and 46% (95% CI: 30–72%), *p* < 0.001 (Figure 2).

**Figure 3:**
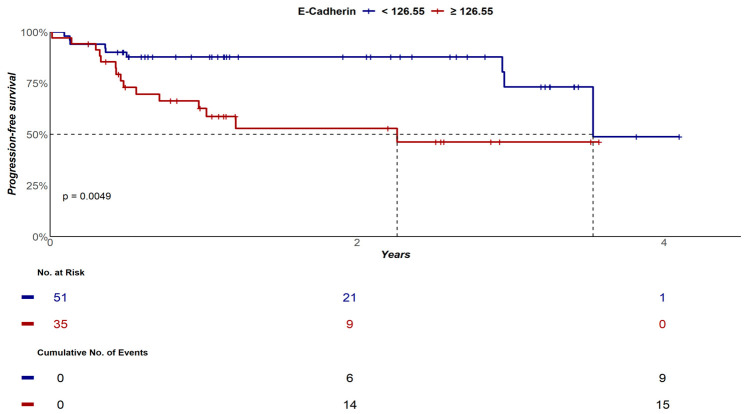
EFS curves of DLBCL patients according to sE-cadherin quantification at diagnosis.

Median EFS for cases with sE-cadherin levels < 126.55 ng/mL was 3.5 years (95% CI: 3.5—not reached), while median EFS for DLBCL patients with sE-cadherin levels ≥ 126.55 ng/mL was 2.3 years (95% CI: 0.9—not reached), *p* = 0.004. EFS probabilities at 2 years and at 4 years for cases with sE-cadherin < 126.55 ng/mL were 88% (95% CI: 79–97%) and 49% (95% CI: 21–100%), respectively; while EFS probabilities at 2 years and at 4 years for patients with sE-cadherin ≥ 126.55 ng/mL were 49% (95% CI: 21–100%) and 46% (95% CI: 30–72%), respectively, *p* = 0.004 (Figure 3).

### 3.5. Correlation Between sCAM Quantification and Response, Relapse and Mortality

Among 87 ND-DLBCL patients included in the study, 22 died (25%), 79 (91%) had response evaluated after first-line therapy, and of these, 86% (68/79) achieved CR. Progression/Relapse occurred in 27.5% (24/87) of cases. Statistically significant differences were demonstrated between median quantification of sE-cadherin in patients who died (*p* = 0.015), who achieved CR (*p* = 0.019), and who had progression/relapse (*p* = 0.016), when compared to cases who did not present these outcomes, as summarized in Table 3, supporting the potential role of sE-cadherin as a new plasma biomarker predictor of death, therapeutic response, and disease recurrence in ND-DLBCL. On the other hand, no statistically significant correlation was observed between plasma levels of sICAM-1, sVCAM-1, and sP-selectin and response to first-line therapy, disease recurrence, and mortality in this DLBCL cohort.

### 3.6. Correlation Between sCAMs Quantification and Clinical-Phenotypic Features

Using the cut-offs for each sCAMs determined by maxstat test, we searched for clinical-biological associations between plasma sCAMs levels and the main clinical-laboratory findings of 87 ND-DLBCL patients using chi-square and Fisher’s exact tests.

Plasma sICAM-1 levels ≥ 942.65 ng/mL were associated with higher IPI categories [82% (9/11) of patients with sICAM-1 ≥ 942.65 ng/mL had IPI 3–5 vs. 43% (32/74) of patients with sICAM-1 < 942.65 ng/mL, *p* = 0.017], higher frequency of B-symptoms [91% (10/11) of patients with sICAM-1 ≥ 942.65 ng/mL had B-symptoms vs. 47% (35/74) of patients with sICAM-1 < 942.65 ng/mL, *p* = 0.007], and higher rates of anemia [91% (10/11) of patients with sICAM-1 ≥ 942.65 ng/mL had hemoglobin < 12 g/dL vs. 41% (30/74) of patients with sICAM-1 < 942.65 ng/mL, *p* = 0.002].

Plasma sVCAM-1 levels ≥ 7001.35 ng/mL were associated with higher frequency of B-symptoms [100% (10/10) of patients with sVCAM-1 ≥ 7001.35 ng/mL had B-symptoms vs. 48% (37/77) of patients with sVCAM-1 < 7001.35 ng/mL, *p* = 0.002], higher frequency of bone marrow involvement by NHL [60% (6/10) of patients with sVCAM-1 ≥ 7001.35 ng/mL had bone marrow infiltration vs. 13% (10/77) of patients with sVCAM-1 < 7001.35 ng/mL, *p* = 0.002], higher rates of anemia [100% (10/10) of patients with sVCAM-1 ≥ 7001.35 ng/mL had hemoglobin < 12 g/dL vs. 39% (30/77) of patients with sVCAM-1 < 7001.35 ng/mL, *p* < 0.001], higher frequency of lymphopenia [60% (6/10) of patients with sVCAM-1 ≥ 7001.35 ng/mL had lymphocyte counts < 1.0 × 10^9^/L vs. 17% (13/77) of patients with sVCAM-1 < 7001.35 ng/mL, *p* = 0.016], as well as higher frequency of LDH elevation [90% (9/10) of patients with sVCAM-1 ≥ 7001.35 ng/mL had baseline LDH levels ≥ 250 U/L vs. 45% (35/77) of patients with sVCAM-1 < 7001.35 ng/mL, *p* = 0.015].

Plasma levels of sP-selectin ≥ 138.37 ng/mL were only associated with higher frequency of B-symptoms [74% (14/19) of patients with sP-selectin ≥ 138.37 ng/mL had B-symptoms vs. 47% (31/66) of patients with sP-selectin < 138.37 ng/mL, *p* = 0.040].

Finally, plasma sE-cadherin levels ≥ 126.55 ng/mL were associated with higher occurrence of intermediate-high and high-risk IPI [63% (22/35) of patients with sE-cadherin ≥ 126.55 ng/mL had IPI 3–5 vs. 39% (20/51) of patients with sE-cadherin < 126.55 ng/mL, *p* = 0.031], higher frequency of B-symptoms [83% (29/35) of patients with sE-cadherin ≥ 126.55 ng/mL had B-symptoms vs. 33% (17/51) of patients with sE-cadherin < 126.55 ng/mL, *p* < 0.001], higher frequency of unfavorable status performance [37% (13/35) of cases with sE-cadherin ≥ 126.55 ng/mL presented ECOG ≥ 2 vs. 14% (7/51) of patients with sE-cadherin < 126.55 ng/mL, *p* = 0.012], higher rates of anemia [71% (25/35) of patients with sE-cadherin ≥ 126.55 ng/mL had hemoglobin < 12 g/dL vs. 29% (15/51) of patients with sE-cadherin < 126.55 ng/mL, *p* < 0.001], as well as higher peripheral blood monocyte counts [median monocyte count = 0.69 × 10^9^/L (IqR 25–75%: 0.52–0.88) for patients with sE-cadherin ≥ 126.55 ng/mL vs. 0.50 × 10^9^/L (IqR 25–75%: 0.41–0.61) for patients with sE-cadherin < 126.55 ng/mL, *p* = 0.019].

### 3.7. Prognostic Factors: Univariate and Multivariate Analyses

In univariate analysis, prognostic factors associated with decreased OS in 87 ND-DLBCL patients were: continuous increases in age [HR: 1.05; 95% CI: 1.02–1.09; *p* = 0.003], advanced-stage disease (Ann Arbor III/IV) [HR: 2.68; 95% CI: 0.90–7.97; *p* = 0.076], intermediate-high/high-risk IPI [HR: 3.37; 95% CI: 1.31–8.65; *p* = 0.012], presence of B-symptoms [HR: 3.61; 95% CI: 1.32–9.85; *p* = 0.012], ECOG ≥ 2 [HR: 5.01; 95% CI: 2.09–12.00; *p* < 0.001], anemia (Hb < 12 g/dL) [HR: 4.56; 95% CI: 1.68–12.42; *p* = 0.003], neutropenia < 1.5 × 10^9^/L [HR: 10.8; 95% CI: 2.25–51.64; *p* = 0.003], lymphopenia < 1.0 × 10^9^/L [HR: 3.22; 95% CI: 1.26–8.33; *p* = 0.015], monocytopenia < 0.1 × 10^9^/L [HR: 18.0; 95% CI: 2.07–56.1; *p* = 0.009]; continuous increases in neutrophil/lymphocyte ratio [HR: 1.09; 95% CI: 1.05–1.14; *p* < 0.001]; continuous increases in sE-cadherin plasma levels [HR: 1.002; 95% CI: 1.000–1.005; *p* = 0.046], sE-cadherin ≥ 126.55 ng/mL [HR: 4.25; 95% CI: 1.70–10.64; *p* = 0.002]; treatment with R-miniCHOP regimen [HR: 15.40; 95% CI: 4.85–48.89; *p* < 0.001], omission of radiotherapy from first-line therapy [HR: 10.19; 95% CI: 1.35–76.82; *p* = 0.024] and administration of cytoreductive chemotherapy [HR: 4.60; 95% CI: 1.87–11.32; *p* < 0.001].

Additionally, factors associated with poor EFS in univariate analysis were: continuous increases in age [HR: 1.05; 95% CI: 1.01–1.09, *p* = 0.003], advanced-stage disease [HR: 3.10; 95% CI: 1.05–9.12; *p* = 0.040], IPI ≥ 3 [HR: 3.95; 95% CI: 1.56–10.02; *p* = 0.004], B-symptoms [HR: 4.24; 95% CI: 1.57–11.48; *p* = 0.004], ECOG ≥ 2 [HR: 4.01; 95% CI: 1.75–9.20; *p* = 0.001], anemia (hemoglobin < 12 g/dL) [HR: 4.12; 95% CI: 1.63–10.44; *p* = 0.003], neutrophil count < 1.5 × 10^9^/L [HR: 9.88; 95% CI: 2.09–46.56; *p* = 0.004], lymphocyte count < 1.0 × 10^9^/L [HR: 2.60; 95% CI: 1.05–6.41; *p* = 0.038], continuous increases in neutrophil/lymphocyte ratio [HR: 1.09; 95% CI: 1.04–1.13; *p* < 0.001], LDH ≥ 250 IU/L [HR: 2.42; 95% CI: 1.02–5.68; *p* = 0.043], continuous increases in sICAM-1 levels [HR: 1.002; 95% CI: 1.000–1.003; *p* = 0.030]; continuous increases in sE-cadherin plasma levels [HR: 1.002; 95% CI: 1.000–1.004; *p* = 0.027], sE-cadherin ≥ 126.55 ng/mL [HR: 3.15; 95% CI: 1.35–7.30; *p* = 0.008], treatment with R-miniCHOP [HR: 11.87; 95% CI: 3.93–35.76; *p* < 0.001], omission of radiotherapy from first-line treatment [HR: 11.68; 95% CI: 1.55–87.84; *p* = 0.017] and use of cytoreductive therapy [HR: 3.50; 95% CI: 1.52–8.01; *p* = 0.003].

In multivariate analysis, independent predictors associated with poor OS were continuous increases in age [HR: 1.048; 95% CI: 1.013–1.084; *p* = 0.007], continuous increases in neutrophil/lymphocyte ratio [HR: 1.051; 95% CI: 1.007–1.098; *p* = 0.024], and sE-cadherin levels ≥ 126.55 ng/mL [HR: 3.51; 95% CI: 1.01–11.21; *p* = 0.034]. Similarly, continuous increases in age [HR: 1.051; 95% CI: 1.016–1.088; *p* = 0.004] and continuous increases in neutrophil/lymphocyte ratio [HR: 1.047; 95% CI: 1.004–1.093; *p* = 0.033] independently predicted worse EFS.

## 4. Discussion

In this study, we demonstrated for the first time that plasma quantification of sE-cadherin by sandwich enzyme-linked immunosorbent assay (ELISA) at diagnosis is able to predict response to R-CHOP immunochemotherapy, disease recurrence or progression, and overall survival in ND-DLBCL. These results highlight the potential role of sE-cadherin as a novel prognostic biomarker in DLBCL.

Cadherins constitute a group of calcium-dependent cell adhesion molecules responsible for maintaining cohesion between cells, preserving normal tissue architecture [15,16]. E-cadherin is a type 1-cadherin that forms homophilic adherent junctions between epithelial cells, maintaining the tissue barrier integrity. E-cadherin has three domains: an extracellular, a transmembrane, and an intracellular. The extracellular domain is formed by five consecutive cadherin repeat subdomains, while the intracellular domain exhibits a functional binding site for β-catenin [22].

Cadherins play a central role in cancer biology, actively participating in tumor invasiveness, dissemination and metastasis [22]. These processes are favored by E-cadherin loss of function, with consequent switching for N-cadherin, which triggers the epithelial–mesenchymal transition of malignant cells [16,22]. Switching of E-cadherin for N-cadherin improves cell migration capacity, increasing its motility and invasiveness, contributing to cancer progression. Inactivation of E-cadherin can result from genetic mutation, such as loss of heterozygosity of chromosome 16q21-22, associated with *CHD1* gene deletion, or can also result from epigenetic silencing through hypermethylation of the *CHD1* promoter region. Both mechanisms are well described in solid tumors, such as breast, gastric, endometrial, ovarian, and thyroid malignant neoplasms [23,24]. Epigenetic disruption substantially contributes to DLBCL oncogenesis, since mutations in epigenetic regulators and dysregulation of specific microRNAs lead to altered chromatin states, repression of tumor suppressor genes, enhanced oncogenic signaling and promote therapeutic resistance, favoring tumor immune evasion [25]. Therapies aiming to promote epigenetic reprogramming in DLBCL, such as tazemetostat, an *EZH2* inhibitor, act targeting epigenetic vulnerabilities, favoring tumor cells differentiation, blocking its proliferation, and inducing a proapoptotic plasma cell-like or memory B cell-like state. This epigenetically programmed identity crisis constitutes a promising therapeutic strategy to target DLBCL [26,27].

Recent studies have shown that E-cadherin tissue loss occurring in different malignancies is associated with increased levels of its soluble form (sE-cadherin) in plasma and other body fluids. This process occurs through proteolytic cleavage of membrane E-cadherin mediated by several proteases, including members of the ADAM family (ADAM-10 and ADAM-15), cathepsins, kallikrein-7, metalloproteinases (MMP-2, MMP-3, MMP-7, MMP-9 and MT1-MMP) and plasmin [28,29,30,31,32]. Tissue loss of E-cadherin reduces cohesion of neoplastic cells, while increase in its soluble fragments reflects intense degradation of cadherin-mediated cell junctions, facilitating occurrence of metastases [32,33,34].

High soluble levels of E-cadherin have been demonstrated in many solid tumors for over a decade. In these malignancies, sE-cadherin has stood out as a biomarker of epithelial tumors, as well as showing an association with disease progression and recurrence [32]. In this sense, elevated sE-cadherin levels have been correlated with higher grade, number of tumors, and relapse in bladder cancer [35], cancer spread in colorectal tumors [36], tumor size, higher recurrence, and poor survival in gastric cancer [37], number of tumors, size, advanced stage, and vascular invasion in liver cancer [38], and biochemical failure predisposing to higher recurrence rates in early-stage prostate cancer [39].

Although widely studied in epithelial malignancies, the role of E-cadherin in hematological malignancies biology, as well as its potential prognostic impact in this setting, remains unclear. Syrigos KN et cols. demonstrated that multiple myeloma patients have sE-cadherin levels five times higher than healthy controls (*p* < 0.001) [40]. Furthermore, sE-cadherin has been shown to be a predictor of survival in multiple myeloma, as patients with serum levels below 3000 ng/mL had prolonged survival, while an increase in sE-cadherin of 100 ng/mL increased the risk of death by 6% [40]. Recently, Hirao M. et cols. showed that negative expression of E-cadherin in the membranes of multiple myeloma cells in bone marrow is associated with occurrence of extramedullary disease [41]. Additionally, Melki JR et cols. demonstrated loss of gene and protein expression of E-cadherin in samples from patients with acute myeloid leukemia and chronic lymphocytic leukemia due to abnormal E-cadherin gene hypermethylation, thus neutralizing its natural function as a “metastasis suppressor” [42].

Currently, there are few studies evaluating the role of E-cadherin in NHLs, and to date there are no reports on its potential impact on prognosis of DLBCL patients. In 2002 Takubo T et cols. quantified sE-cadherin levels in 30 NHL patients and evaluated the tissue expression of this biomolecule by immunohistochemistry in only 3/15 patients presenting sE-cadherin levels over mean + 2SD. The results indicated that E-cadherin antigen was expressed in lymphoma cells of the lymph nodes and that sE-cadherin might be released into the blood from lymphoma cells. However, this study presented numerous methodological limitations, including limited and heterogeneous sampling, not allowing any robust conclusions to be drawn [43]. Eissa LA et cols. also quantified the molecules E-cadherin, GAG, and MDA in 40 ND-DLBCL patients. Although serum levels of the three biomolecules were significantly higher in DLBCL compared to healthy controls (*p* < 0.05), no biological-phenotypic or prognostic association could be established [44].

Our study was the first to establish association between elevated serum levels of sE-cadherin and higher rates of chemoresistance to R-CHOP therapy in DLBCL. Here, patients with higher sE-cadherin levels experienced higher rates of treatment failure (*p* = 0.019) and recurrence (*p* = 0.016). Since publication of the POLARIX study results, R-CHP plus polatuzumab-vedotin has become the new gold standard for first-line therapy of DLBCL, NOS presenting non-GCB phenotype, advanced clinical stage and intermediate-high or high-risk IPI, conferring an increase in PFS compared to the R-CHOP regimen [45]. Our study determined the role of sE-cadherin as a potential biomarker able to predict response in DLBCL patients treated exclusively with R-CHOP and R-CHOP-like regimens. We should emphasize that the role of plasma quantification of sE-cadherin has not been addressed in light of the novel treatment regimens based on polatuzumab-vedotin and should be tested in a near future in populations treated with first-line regimens containing this drug.

Similarly, ND-DLBCL patients presenting sE-cadherin ≥ 126.55 ng/mL independently had 3.5 times greater chance of death than patients containing plasma levels below this cutoff point [HR: 3.51; 95% CI: 1.01–11.21; *p* = 0.034]. We also demonstrated significant association between high sE-cadherin levels at diagnosis with several unfavorable clinical-phenotypic findings, including more advanced IPI categories (3–5), higher frequency of constitutional symptoms, poorer performance status, higher rates of anemia and higher peripheral blood monocyte counts. All of these factors are classically known as markers of adverse prognosis in NHL [7,46,47,48,49].

Due to DLBCL prognostic heterogeneity, identification of plasma markers associated with cancer biology is of great importance to aid in its prognostic discrimination. Many biomarkers presenting prognostic impact have little utility in clinical practice, constituting genetic-molecular alterations that require complex, costly and not readily available methodologies. Many of these techniques are difficult to interpret, and are not available outside research settings. Therefore, identification of sE-cadherin as a potential novel prognostic biomarker in ND-DLBCL adds valuable and practical information. Quantification of sE-cadherin by ELISA offers a number of operational and technical benefits, including processing speed, analyte stability, low cost, wide availability, high reproducibility, use of easily obtainable samples (peripheral blood), and rapid results [50,51].

Identification of sE-cadherin as an important biomarker associated with DLBCL biology also expands the potential for therapeutic opportunities aimed at controlling this tumor. In solid tumors, particularly gastric adenocarcinoma, where E-cadherin dysfunction is frequent, some drugs have been used experimentally aiming reconstitute E-cadherin tissue expression or interfering with E-cadherin downstream molecules. In this sense, considering the high frequency of *CDH1* promoter region hypermethylation, epigenetic modifiers, such as hypomethylating agents (5-azacytidine or decitabine) or histone deacetylase inhibitors (vorinostat, belinostat, romidepsin), have theoretical potential to add benefit to the therapy of DLBCL patients exhibiting high sE-cadherin levels. Additionally, the use of drugs that interfere with HER receptors and NOTCH pathway could be considered for experimental tests, as they act in E-cadherin-mediated signaling [52]. Currently, several drugs, including the monoclonal antibodies 66E8 and 19A11, which target the ectodomains of E-cadherin, resveratrol (increases *CDH1* histone acetylation), atovaquone (promotes E-cadherin expression by targeting PDGFRβ/NF-kB signaling pathway), pirfenidone (upregulates E-cadherin expression by inhibiting Smad2/3 signaling through TGF-β inhibition), among many other natural compounds, have been used experimentally in various cancers targeting E-cadherin [53].

Although prospective, with detailed clinical and laboratory data retrieval and involving a relatively homogeneous population (>95% of DLBCL, NOS), our study presents some limitations. Among these, we highlight the relatively small sample size, its single-center nature, the short follow-up time, and the absence of E-cadherin tissue protein expression analysis in lymph nodes involved by DLBCL. This last analysis would allow to establish whether, in tumors of non-epithelial lineage, the source of sE-cadherin comes from the tumor cells themselves or, instead, results from the tumor invasion of lymphoid B-cells into epithelial tissues. Another limitation is the absence of an independent external validation cohort. Although the prospective design and homogeneous treatment approach strengthen the robustness of our findings, external validation in larger and multicenter populations, containing longer follow-up time, is essential to confirm our results.

## 5. Conclusions

In conclusion, although plasma quantification of other sCAMs, such as sICAM-1, sVCAM-1, and sP-selectin did not demonstrate prognostic impact in DLBCL, this study established in a pioneering way the role of sE-cadherin as a novel prognostic biomarker in DLBCL, able to predict response to R-CHOP therapy, relapse, and association with unfavorable clinical-phenotypic features. Additionally, high plasma levels of sE-cadherin at diagnosis were able to independently predict decreased OS in ND-DLBCL and may represent a potential new target for directed therapy.

## Figures and Tables

**Table 1:** Clinical-demographic and pathological features of the 87 DLBCL patients.

Characteristic	Absolute Value (*N*) [*N* = 87]	Relative Value (%)
Age	Median: 63 years	IqR 25–75%: 51–69 years
Gender	Female: 46	53%
	Male: 41	47%
Histological classification	DLBCL, NOS: 83	95.4%
	DLBCL rich T/H: 2	2.4%
	DLBCL, NOS EBV+: 1	1.1%
	DLBCL, leg-type: 1	1.1%
COO by Hans algorithm	GCB: 48	55%
	Non-GCB: 39	45%
Ann Arbor CS	I or II: 31	36%
	III or IV: 56	64%
Extranodal involvement	72	83%
≥2 extranodal sites	39	45%
Bulky disease ≥ 7 cm	41	47%
B-symptoms	47	54%
Bone marrow infiltration	16	19%
CNS involvement	3	3.4%
ECOG ≥ 2	20	23%
IPI categorization	Low-risk: 23	26%
	Low-intermediate: 22	25%
	High-intermediate: 21	24%
	High-risk: 21	24%
R-IPI categorization	Very good: 6	7%
	Good: 39	45%
	Poor: 42	48%

Abbreviations: DLBCL, NOS: diffuse large B-cell lymphoma, not otherwise specified; DLBCL rich T/H: diffuse large B-cell lymphoma rich in T-cells/histiocytes; DLBCL, NOS EBV+: diffuse large B-cell lymphoma, not otherwise specified Epstein–Barr virus-associated; COO: cell of origin; GCB: germinal center B-cell; non-GCB: non-germinal center B-cell phenotype; CS: clinical stage; CNS: central nervous system; ECOG: Eastern Cooperative Oncology Group performance status classification; IPI: International Prognostic Index; R-IPI: Revised International Prognostic Index.

**Table 2:** Baseline laboratory features of 87 DLBCL patients presented as categorical variables.

Laboratory Features	N	%
Hemoglobin		
<12 g/dL	40	46%
≥12 g/dL	47	54%
WBC		
<4.0 × 10^9^/L	4	5%
4.0–11.0 × 10^9^/L	67	77%
>11 × 10^9^/L	16	18%
Neutrophils		
<1.5 × 10^9^/L	2	2%
1.5–7.0 × 10^9^/L	66	76%
>7.0 × 10^9^/L	19	22%
Lymphocytes		
<1.0 × 10^9^/L	19	22%
1.0–3.0 × 10^9^/L	60	69%
>3.0 × 10^9^/L	8	9%
Monocytes		
<0.1 × 10^9^/L	1	1%
0.1–1.0 × 10^9^/L	75	86%
>1.0 × 10^9^/L	11	13%
Platelets		
<150 × 10^9^/L	5	5%
150–400 × 10^9^/L	64	74%
>400 × 10^9^/L	18	21%
LDH		
<UNV	43	49%
≥UNV	44	51%

Abbreviations: WBC: white blood count; LDH: lactate dehydrogenase; UNV: upper normal value.

**Table 3:** Association between sE-cadherin levels and Response, Relapse and Mortality in DLBCL.

	sE-Cadherin Median	sE-Cadherin Median	*p*-Value
Response (CR)	Achieved CR (*n* = 68)	PR/SD/PD (*n* = 11)	0.019
	102 ng/mL	135 ng/mL	
	(IqR 25–75%: 50–159)	(IqR 25–75%: 117–217)	
Relapse/Progression	Without R/P (*n* = 63)	With R/P (*n* = 24)	0.016
	101 ng/mL	136 ng/mL	
	(IqR 25–75%: 48–142)	(IqR 25–75%: 111–240)	
Mortality	Survivors (*n* = 65)	Deceased (*n* = 22)	0.015
	102 ng/mL	145 ng/mL	
	(IqR 25–75%: 49–141)	(IqR 25–75%: 113–263)	

Abbreviations: sE-cadherin: soluble E-cadherin quantification; CR: complete response; PR: partial response; SD: stable disease; PD: progression of disease; R/P: relapse/progression of disease. Comparison made by Wilcoxon test with Bonferroni adjustment.

## Data Availability

The original contributions presented in this study are included in the article. Further inquiries can be directed to the corresponding author.
